# C-type natriuretic peptide regulates lipid metabolism through a NPRB-PPAR pathway in the intramuscular and subcutaneous adipocytes in chickens

**DOI:** 10.1038/s41598-025-86433-w

**Published:** 2025-04-15

**Authors:** Huayun Huang, Longzhou Liu, Zhong Liang, Qianbao Wang, Chunmiao Li, Zhengyang Huang, Zhenhua Zhao, Wei Han

**Affiliations:** 1 Institute of Poultry Science, Chinese Academy of Agriculture Sciences, 225125 Jiangsu, P. R. China; 2 College of Animal Science, Yangtze University, Jingzhou, 8060550 P. R. China

**Keywords:** C-type natriuretic peptide, Intramuscular adipocytes, Subcutaneous adipocytes, PPAR pathway, Molecular biology, Transcriptomics

## Abstract

Natriuretic peptides (NPs) have an important role in lipid metabolism in skeletal muscle and adipose tissue in animals. C-type natriuretic peptide (CNP) is an important NP, but the molecular mechanisms that underlie its activity are not completely understood. Treatment of intramuscular fat (IMF) and subcutaneous fat (SCF) adipocytes with CNP led to decreased differentiation, promoted proliferation and lipolysis, and increased the expression of natriuretic peptide receptor B (*NPRB*) mRNA. Silencing natriuretic peptide C (*NPPC*) had the opposite results in IMF and SCF adipocytes. Transcriptome analysis found 665 differentially expressed genes (DEGs) in IMF adipocytes and 991 in SCF adipocytes. Seven genes in IMF adipocytes (*FABP4*, *APOA1*, *ACOX2*, *ADIPOQ*, *CD36*, *FABP5*, and *LPL*) and eight genes in SCF adipocytes (*ACOX3*, *ACSL1*, *APOA1*, *CPT1A*, *CPT2*, *FABP4*, *PDPK1* and *PPARα*) are related to fat metabolism. Fifteen genes were found to be enriched in the peroxisome proliferator-activated receptor (PPAR) pathway. Integrated analysis identified 113 intersection genes in IMF and SCF adipocytes, two of which (*APOA1* and *FABP4*) were enriched in the PPAR pathway. In conclusion, CNP may regulated lipid metabolism through the *NPRB*-PPAR pathway in both IMF and SCF adipocytes, *FABP4* and *APOA1* may be the key genes that mediated CNP regulation of fat deposition.

**Supplementary Information:**

The online version contains supplementary material available at 10.1038/s41598-025-86433-w.

## Background and summary

Subcutaneous fat (SCF), abdominal fat (AbF), and intramuscular fat (IMF) are present in poultry. IMF content is associated with tenderness, flavor, and juiciness^1^. Excess SCF and abdominal fat are discarded during slaughtering and processing and have been reported to cause environmental pollution^2^. The contribution of SCF deposition is generally not considered in broiler production, and for that reason, high IMF and low SCF deposition are the main objectives of broiler breeding. Study of the differences in the molecular activity resulting in IMF or SCF deposition would have important theoretical and practical significance.

Hormones are bioactive substances that regulate growth and development, and they also regulate lipid metabolism. Thyroid hormones directly or indirectly stimulate hepatic lipid synthesis and degradation and help to maintain lipid metabolism and energy balance^3^. Growth hormone stimulates adipogenesis in 3T3-L1 cells^4^. Follicle stimulating hormone (FSH) has been shown to promote the differentiation of abdominal adipocytes^5^, and enhance IMF content in breast muscle^6^. Adiponectin^7^and estrogen^8^ were found to decrease fat deposition in chickens. Different hormones have different roles in the regulation of lipid metabolism.

Natriuretic peptides (NPs) are peptide hormones, including atrial natriuretic peptide (ANP)^9^, brain natriuretic peptide (BNP)^10^, and C-type natriuretic peptide (CNP)^11^in mammals, and BNP, CNP and VNP in poultry^12^. NPs act by binding to specific natriuretic peptide receptor A (*NPRA*) and natriuretic peptide receptor B (*NPRB*)^13^and have different effects on adipose tissue and skeletal muscle metabolism. ANP and BNP were reported to promote lipolysis in human fat cells^14^, control brown fat thermogenesis in mice^15^, and participate in mitochondrial oxidative metabolism and fat oxidation in human skeletal muscle myotubes^16^. CNP was shown to regulate food intake and energy expenditure in mice^17^, and overexpression in endothelial cells increased energy expenditure and improved insulin sensitivity and hepatic lipid metabolism in a high-fat diet-induced obesity model^18^. The results of previous studies show that NPs are involved in lipid metabolism in adipose tissue and skeletal muscle in mammals.

BNP stimulates lipolysis in the abdominal adipocytes of chickens by upregulating the expression of its receptor NPRA^19^, but the relationship between CNP and lipid metabolism is not clear. The evidence of studies in mammals suggests that CNP would be expected to regulate lipid metabolism in adipose tissue and skeletal muscle in chickens. This study aimed to clarify the effect of CNP on lipid metabolism in chickens and the molecular mechanisms underlying its activity in adipose tissue and skeletal muscle. Study of differences in the molecular pathways of CNP in lipid metabolism in adipose and skeletal muscle tissues would add to our understanding of the molecular mechanisms of NP regulation of lipid metabolism and provide important theoretical guidance for the breeding of high-quality broilers.

## Methods

### Preadipocyte culture

SCF preadipocytes were isolated from S3 strain chickens (Institute of Poultry Science, Jiangsu, China) at 7–10 days of age as previously described^20^. This strain has yellow feathers, green feet, good meat quality and excellent production performance. Cells were cultured in 96-well and 6-well culture dishes in Dulbecco’s Modified Eagle Medium (DMEM)/F12 (1:1) containing 10% fetal bovine serum and 1% penicillin/streptomycin at 37 °C in a humidified atmosphere of 5% CO_2_in air. When the cells reached 70% confluence, differentiation was induced with oleic acid (305 µmol/L). IMF preadipocytes were isolated from breast muscle tissue of female chickens of the same strain and age as previously described^21^. The method of cell culture was the same as for that of SCF adipocytes.

## CNP treatment

Chicken CNP (Phoenix Pharmaceuticals, Burlingame, CA USA), was added to the culture media at concentrations of 10^−6^, 10^−7^ and 10^−8^ mol/L on day 0. The control group was (DMEM)/F12 (1:1) containing 10% fetal bovine serum and 1% penicillin/streptomycin. Based on phenotype data from the preliminary experiment, 10^−7^ mol/L was determined as the optimal concentration (the data is not shown). Cell proliferation was assayed with a Cell Counting Kit-8 (CCK-8; Dojindo, Kyushu, Japan) and 5-ethynyl-2’-deoxyuridine (EdU) staining (Ruibo, Guangzhou, China) in eight replicate wells, with three replicate wells for each treatment. Adipogenesis was monitored by morphologic examination of the cellular accumulation of lipid droplets by Oil Red O staining. On days 3 and 6, adipocytes were fixed with 10% formaldehyde, washed with phosphate buffered saline, and stained with Oil Red O (0.3% in 60% isopropanol) followed by extensive washes. Stained triglyceride droplets were visualized and photographed.

### *NPPC* siRNA synthesis and transfection

The *NPPC* siRNA sequence was synthesized by GenePharma (Shanghai GenePharma, Shanghai, China). According to the mRNA sequence of chicken *NPPC* (NC_006108.3), three pairs of siRNA primers were designed with Oligo Designer version 3.0 (Shanghai GenePharma) (Table 1), and the *NPPC*−221 primer was validated in vitro.

Preadipocytes were plated on 12-well or 96-well plates in DMEM/F12 without antibiotics. Transfection was performed at 70–80% confluency (Qiagen, Düsseldorf, Germany). Adipocytes were cultured in 12-well plates and transfected with *NPPC*−221 siRNA (1,000 nmol per well) before evaluating gene expression by quantitative real-time polymerase chain reaction (qPCR) and cell differentiation by Oil Red O staining. After transfection with *NPPC*−221 siRNA (250 nmol per well), proliferation was monitored in each study group by CCK8 assays of cells growing in 96-well plates.

**Table 1:** *NPPC* siRNA primers.

Primer name	Sense(5’−3’)	Antisense(5’−3’)
NPPC-Gallus-68	GGCAAUGGCCAAACCUAUUTT	AAUAGGUUUGGCCAUUGCCTT
NPPC-Gallus-110	GCUGUUAGAUGAGGAGCUGTT	CAGCUCCUCAUCUAACAGCTT
NPPC-Gallus-221	GACCCGAAACACCAGAGAUTT	AUCUCUGGUGUUUCGGGUCTT

## Lipolysis

Chicken CNP (10^−7^ mol/L) was added to the media, and the differentiated adipocytes were incubated for an additional 6 days. The glycerol concentration in the medium was determined following each treatment in three replicate wells using a commercially available assay kit (Jiancheng, Nanjing, China).

## RNA extraction

Total RNA was isolated from adipocytes using extraction kits (Qiagen, Düsseldorf, Germany) following the manufacturer’s instructions. An ND-1000 spectrophotometer (Agilent Technologies, Palo Alto, CA, USA) was used to measure the concentration and purity of the RNA (A_260:280_ ≥ 1.8 and ≤ 2.0). RNA integrity (RIN ≥ 7) was evaluated with a 2100 Bioanalyzer Lab-on-Chip system (Agilent Technologies). RNA samples were stored at − 80 °C until used.

## mRNA sequencing

Total RNA from SCF and IMF adipocytes treated with and without 10^−7^mol/L CNP for 6 days was used for mRNA sequencing in three replicate wells for each treatment. Data were analyzed as previously described^19^. Fragments per kilobase of exon per million mapped reads (FPKM) was used to quantify gene expression. Differentially expressed genes (DEGs) all had *p*-values ≤ 0.05 and fold changes ≥ 1.5. Significantly enriched pathways were analyzed by Kobas v3.0 (kobas.cbi.pku.edu.cn/help.do) as described by Mao et al.^22^ and Wu et al.^23^. Pathways with < 3 known chicken genes were discarded.

### qPCR of genes

Gene expression was quantified by qPCR (Qiagen, Düsseldorf, Germany). The final concentration of each primer (Table 2) was 10 µmol/µL.

**Table 2:** qPCR primers.

Gene	Sequence (5’ to 3’)
*CPT2*	F: TTATCCGGTTTGTGCCTTCGT
	R: ATTCCCCTTTCTCAGTACCAGT
*PDPK1*	F: AAATCTGCCTGTAAAAGCTCT
	R: AGCCTAATCTTTTGGTAGCATC
*PPARA*	F: CCATTACGGAGTACATGCTT
	R: AAAGGCACTTCTGAAAACGA
*LPL*	F: GGTGACCTGCTTATGCTA
	R: ATATTGCTGCCTCTTCTC
*ACOX2*	F: CATGCTTCGTGCCTTTCGACT
	R: CCCTTCATTGTGCAGATCCG
*ADIPOQ*	F: GCACGTACTTCTTTGCCTACCAC
	R: GTTGCCCTCCCCGTACACC
*CD36*	F: ATTGCATATGATAATTGGCTTG
	R: CATGTACGATATTGTGCCAT
*FABP5*	F: TGTGAAAACGGAAAGCACCT
	R: CTGCTCCTGGATCAATGACC
*FABP4*	F: TGGCCGTGAAGATGTTGAAAG
	R: CGCAGGTATTCCCGAAGGTTG
*APOA1*	F: GGACCAGTTCTCCGCCAAGT
	R: TGCACCAGAGGCGTCATC
*ACOX3*	F: TTACTGTCGATCATTAGCCAT
	R: CATAGCCACCTTGATAGAGC
*ACSL1*	F: CAAAGGAGAAGGTGAGGTGTG
	R: CTTCAACGTACCGTTTGGTAG
*CPT1A*	F: ATGTTTAATACCTCCCGCATC
	R: CCTATCTCCTGCAGTAAGAGC
*NPPC*	F: ACTTCTTTTTTGCCCTGGATTCTTC
	R: CTACTGGGATTGAGCCATCTTGC

### Statistical analysis

Significant differences were assessed by Student’s *t*-test using SPSS, version 19.0 (IBM Corp; Armonk, USA). *P* < 0.05 was considered significant.

## Data Availability

The datasets used and/or analyzed in this study are available from the corresponding author on reasonable request.

### Data records

#### Effect of CNP on lipid metabolism in IMF adipocytes

**Fig. 1:**
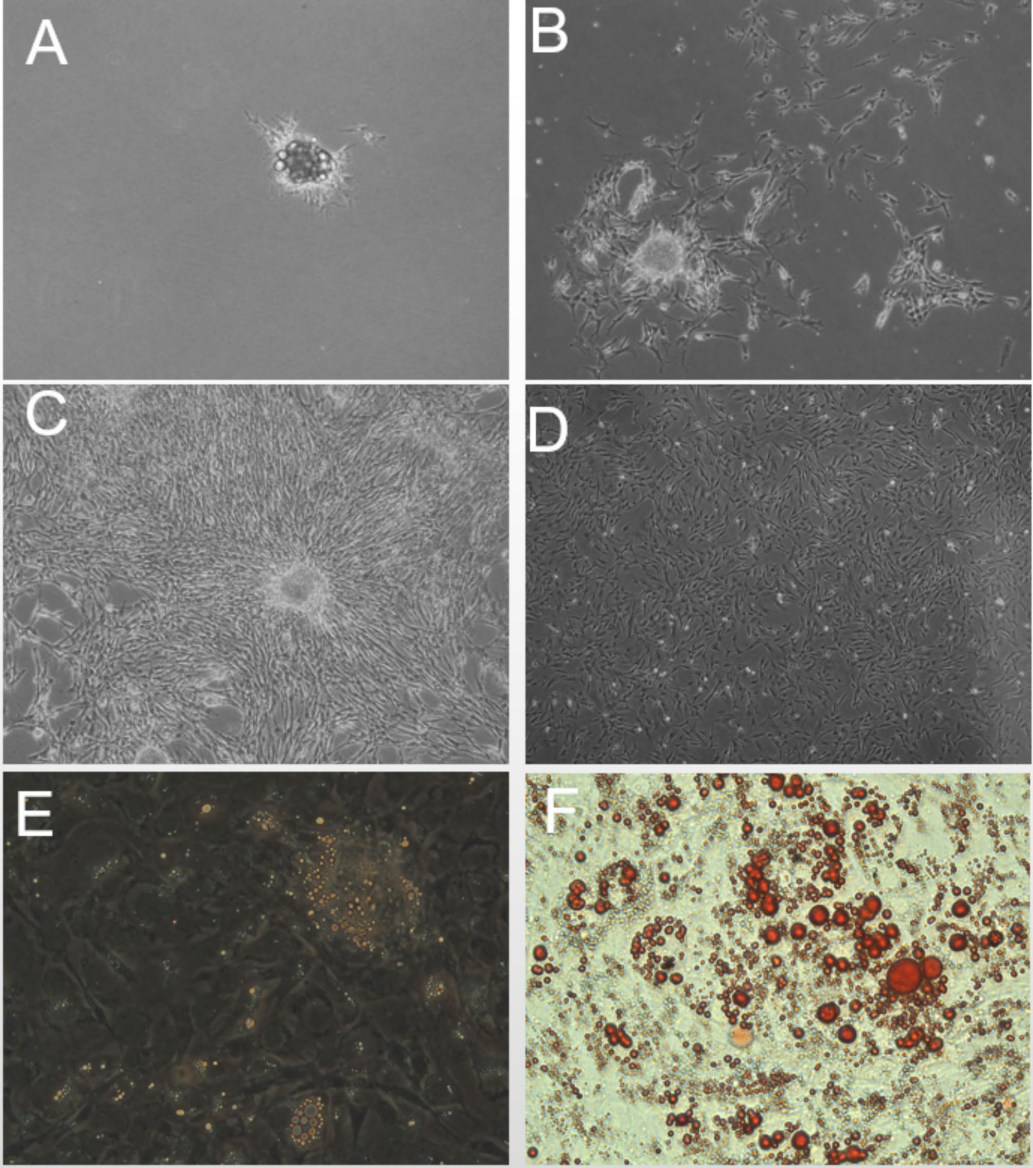
Morphological changes of intramuscular preadipocytes (inverted microscope, 100×). (**A–C**) Cellular morphology of IMF preadipocytes 2, 5, and 7 days after isolation. (**D**) Morphology of IMF adipocytes 24 h after passage. (**E**) Differentiation of IMF adipocytes cultured with oleic acid for 3 days. (**F**) IMF adipocytes stained with Oil red O after induced differentiation for 3 days.

**IMF adipocyte cell morphology** Adipocytes isolated from breast muscle were cultured for 2 days in T25 flasks and evaluated with an inverted microscope. Mature adipocytes began to cluster, a few spindle cells appeared, and some mature adipocytes began to dedifferentiate (Fig. 1A). On day 5, the number of prespindle adipocytes had increased (Fig. 1B); on day 7, the dedifferentiated cells proliferated abundantly (Fig. 1C) and were subcultured. The rate of cell proliferation was high (Fig. 1D). Lipid droplets were clearly visible after induced differentiation for 3 days (Fig. 1E), and large, round, red lipid droplets were visible 6 days after differentiation induced by oleic acid, confirming that the cells were IMF adipocytes (Fig. 1F).

### CNP induction enhanced IMF preadipocyte proliferation, inhibited adipocyte differentiation, and promoted lipolysis

**Fig. 2:**
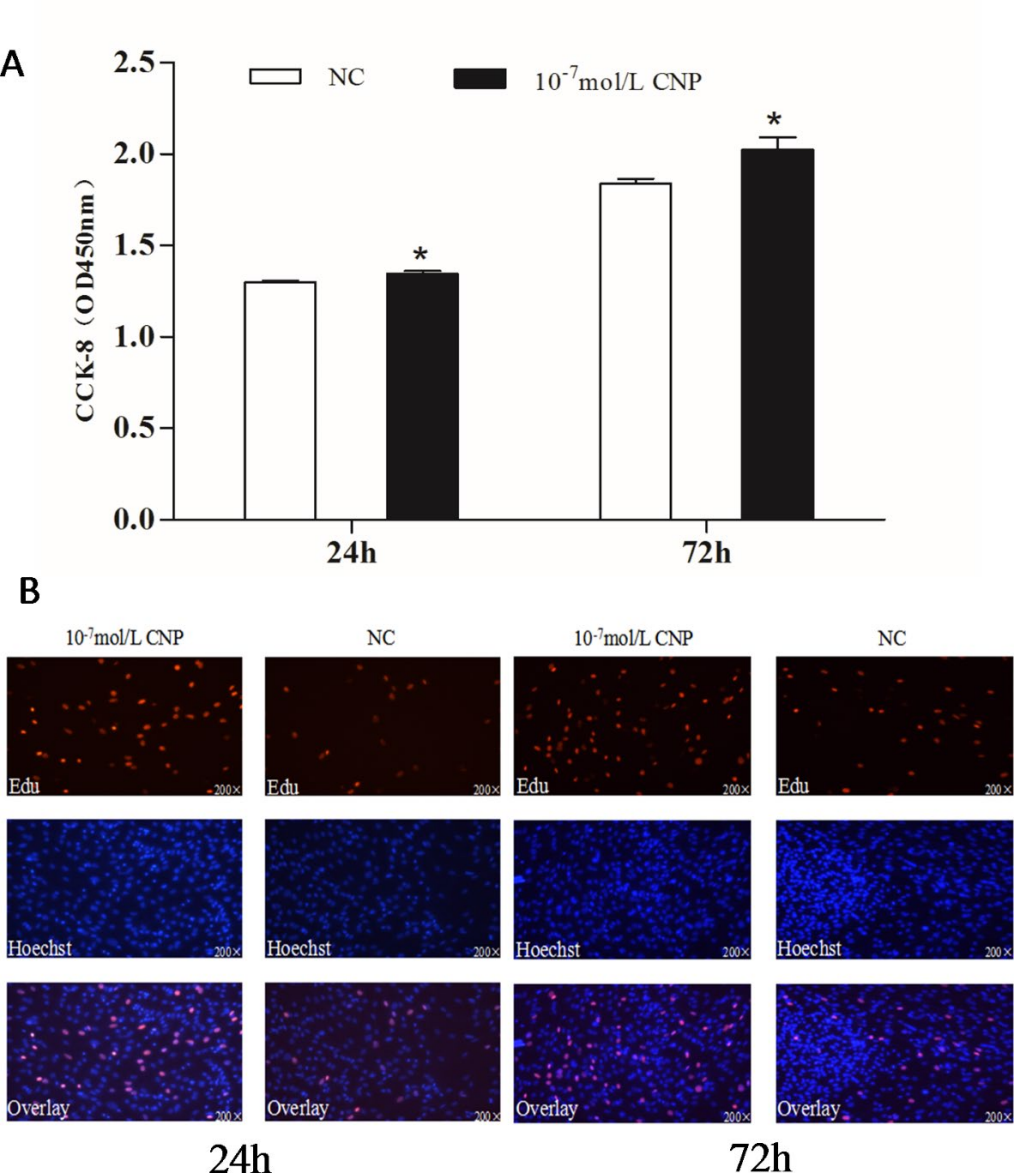
Effect of CNP on the proliferation of intramuscular preadipocyte. (**A**) The number of preadipocytes was significantly increased by CNP (Cell Counting Kit-8, *n* = 8, *p < 0.05 vs. control group). (**B**) EdU staining of intramuscular preadipocytes induced by CNP for 24 h and 72 h (*n* = 3). EdU, number of proliferative cells; Hoechst, number of cells before proliferation; Overlay, superimposition of the two images shows the location of proliferating cells.

**Fig. 3:**
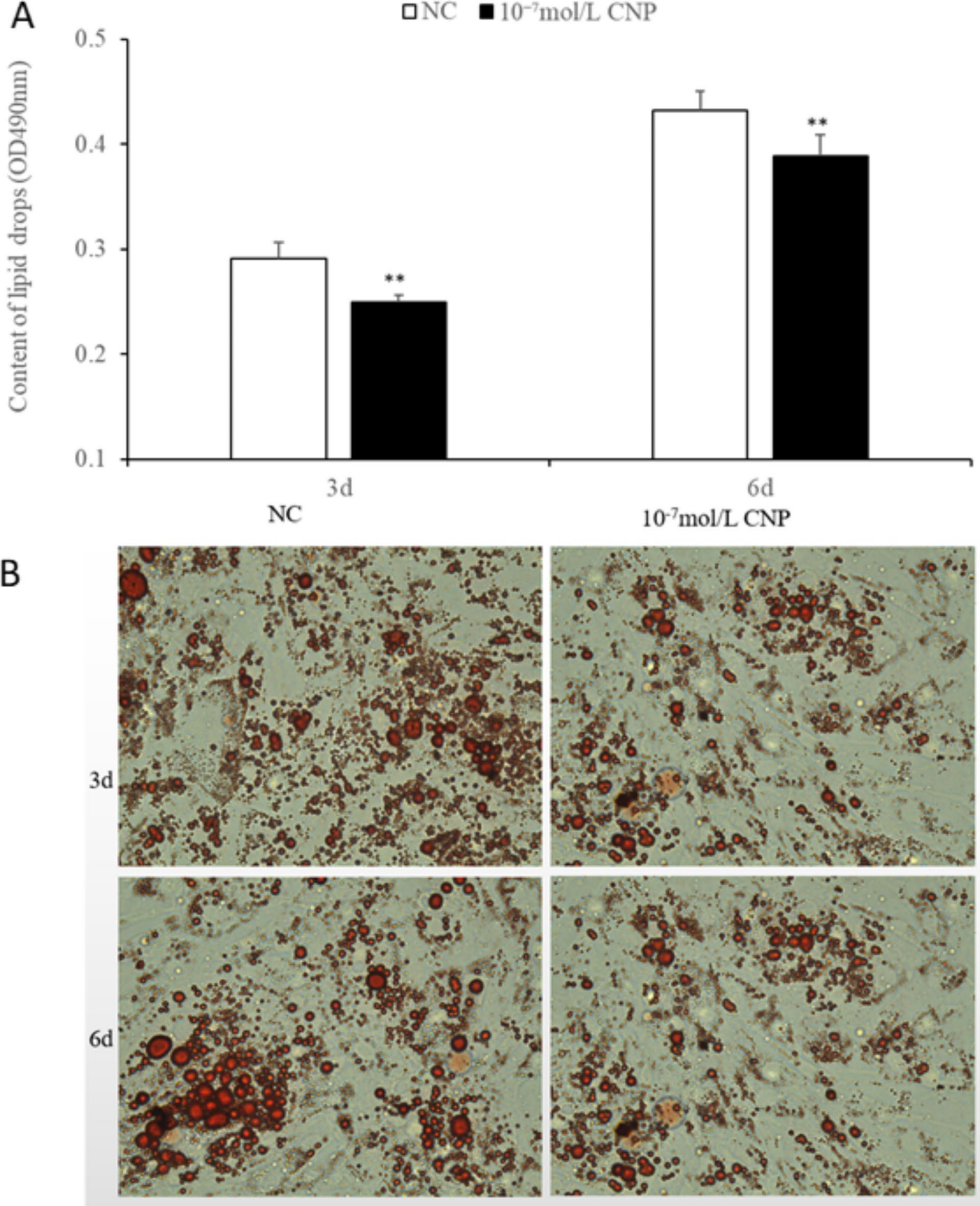
Effect of CNP on differentiation of intramuscular adipocytes. (**A**) CNP 10^−7^ mol/L significantly decreased the differentiation of adipocytes (*n* = 3; **p < 0.01 vs. control). (**B**) Morphologic changes and lipid deposition induced by 10^−7^ mol/L CNP in adipocytes in vitro (inverted microscope, 400× magnification). Oil Red O staining shows that CNP 10^−7^ mol/L decreased both the size and number of the droplets in each accumulation compared with cells not treated with CNP.

**Fig. 4:**
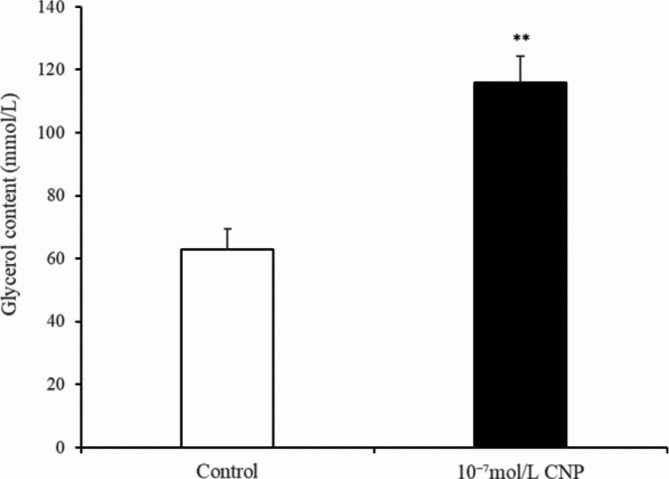
Change of the glycerol content at 6 days after CNP treatment in intramuscular adipocytes (*n* = 3), (**p < 0.01 vs. control).

The number of preadipocytes increased significantly (*p* < 0.05) after 24 and 72 h of culture with 10^−7^ mol/L CNP (Fig. 2A). EdU staining also confirmed that the proliferation rate of CNP-induced cells was increased compared with control cells (Fig. 2B). Differentiated adipocytes were treated with exogenous 10^−7^ mol/L CNP for 3 and 6 d. As shown in Fig. 3A, adipocyte differentiation decreased significantly on treatment with 10^−7^ mol/L CNP (*p* < 0.05). Oil Red O staining showed that the lipid droplets were slightly smaller and fewer in number in cells treated with 10^−7^ mol/L CNP compared with controls (Fig. 3B) Lipolysis was evaluated by measuring glycerol levels in the culture medium. The effects of CNP treatment on the lipolysis of adipocytes are shown in Fig. 4. CNP treatment significantly enhanced glycerol levels compared with those in control cultures at 6 d (*p* < 0.05).

### *NPPC* interference reduced cell proliferation, enhanced adipocyte differentiation and reduced lipolysis in IMF adipocytes

**Fig. 5:**
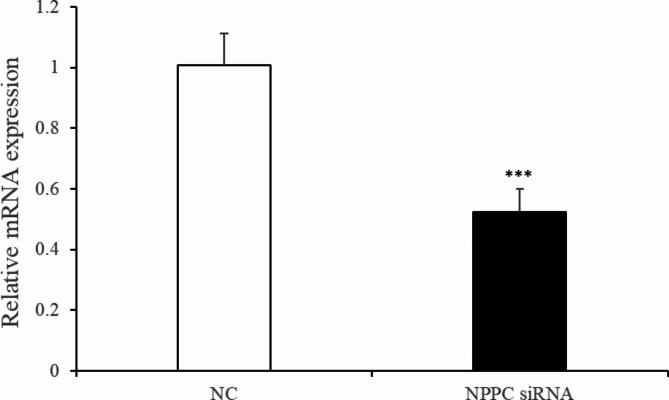
Change of *NPPC* mRNA expression after 24 h transfection with *NPPC* siRNA in the intramuscular adipocytes.

**Fig. 6:**
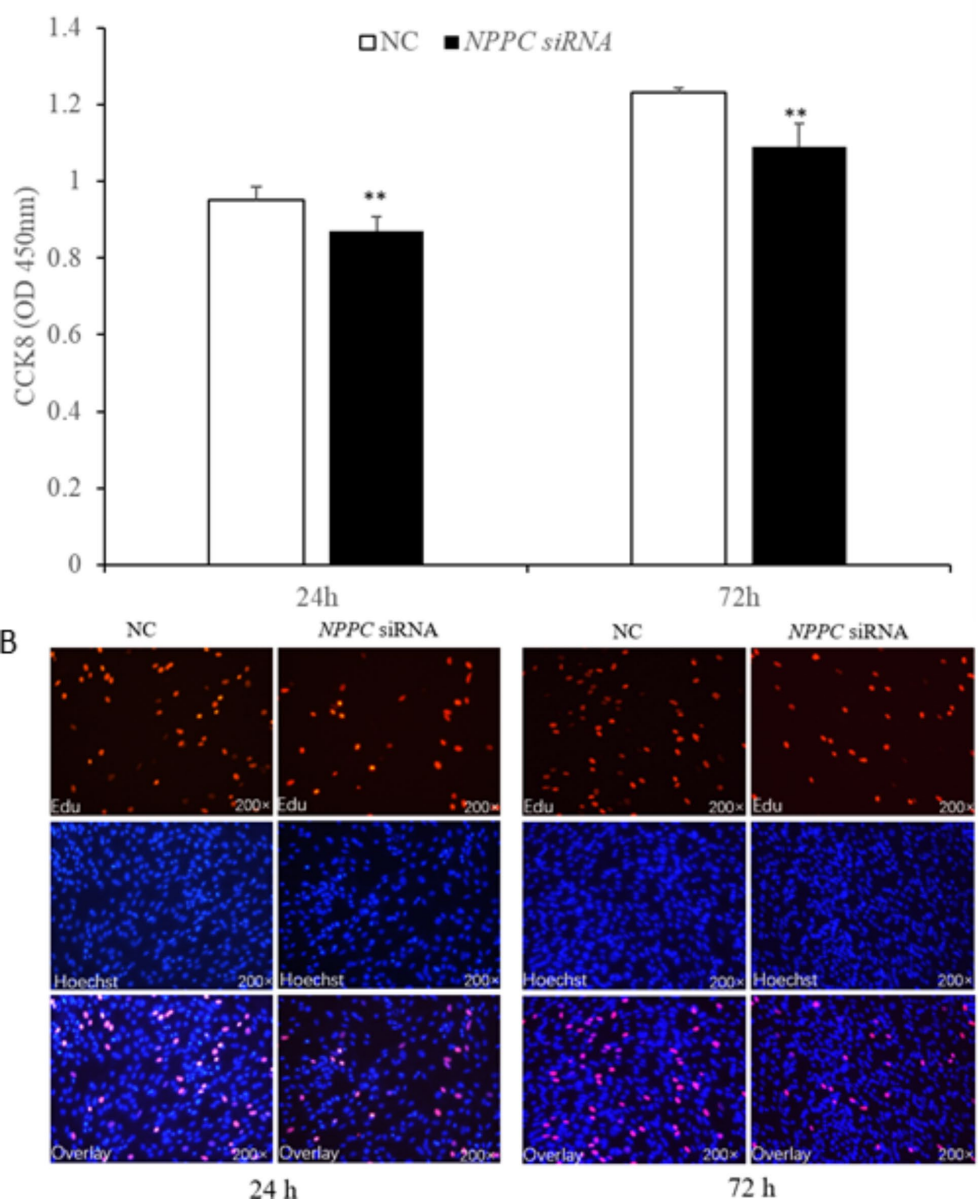
Effect of *NPPC* interference on the proliferation of intramuscular preadipocytes. (**A**) Number of preadipocytes after transfection with *NPPC* siRNA (CCK8 kit, *n* = 8, *p < 0.05 vs. control group).);** B**. EdU staining of chicken preadipocytes after transfection with *NPPC* siRNA. EdU, number of proliferative cells; Hoechst, number of cells before proliferation; Overlay, superimposition of the two images shows the location of proliferating cells.

**Fig. 7:**
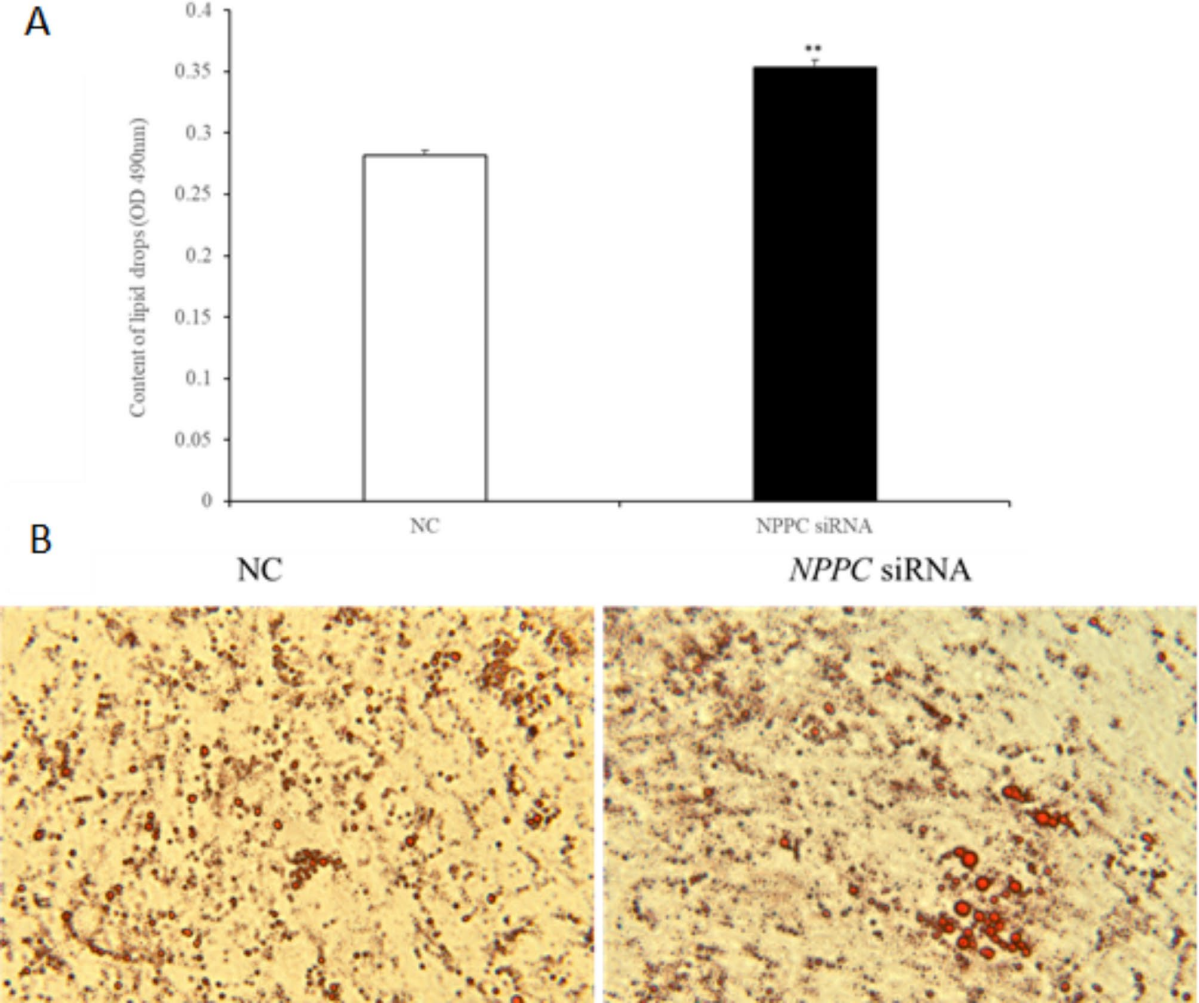
Effect of *NPPC* interference on the differentiation of intramuscular adipocytes. (**A**) Adipocyte differentiation increased significantly after transfection with *NPPC* siRNA (*n* = 3) (**p < 0.01 vs. control). (**B**) Morphologic changes and lipid deposition after transfection with *NPPC* siRNA in adipocytes in vitro (inverted microscope, 400× magnification). Lipid droplets (stained with Oil Red O) accumulated as larger clusters and in greater numbers in cells transfected with *NPPC* siRNA compared with the negative control vector.

**Fig. 8:**
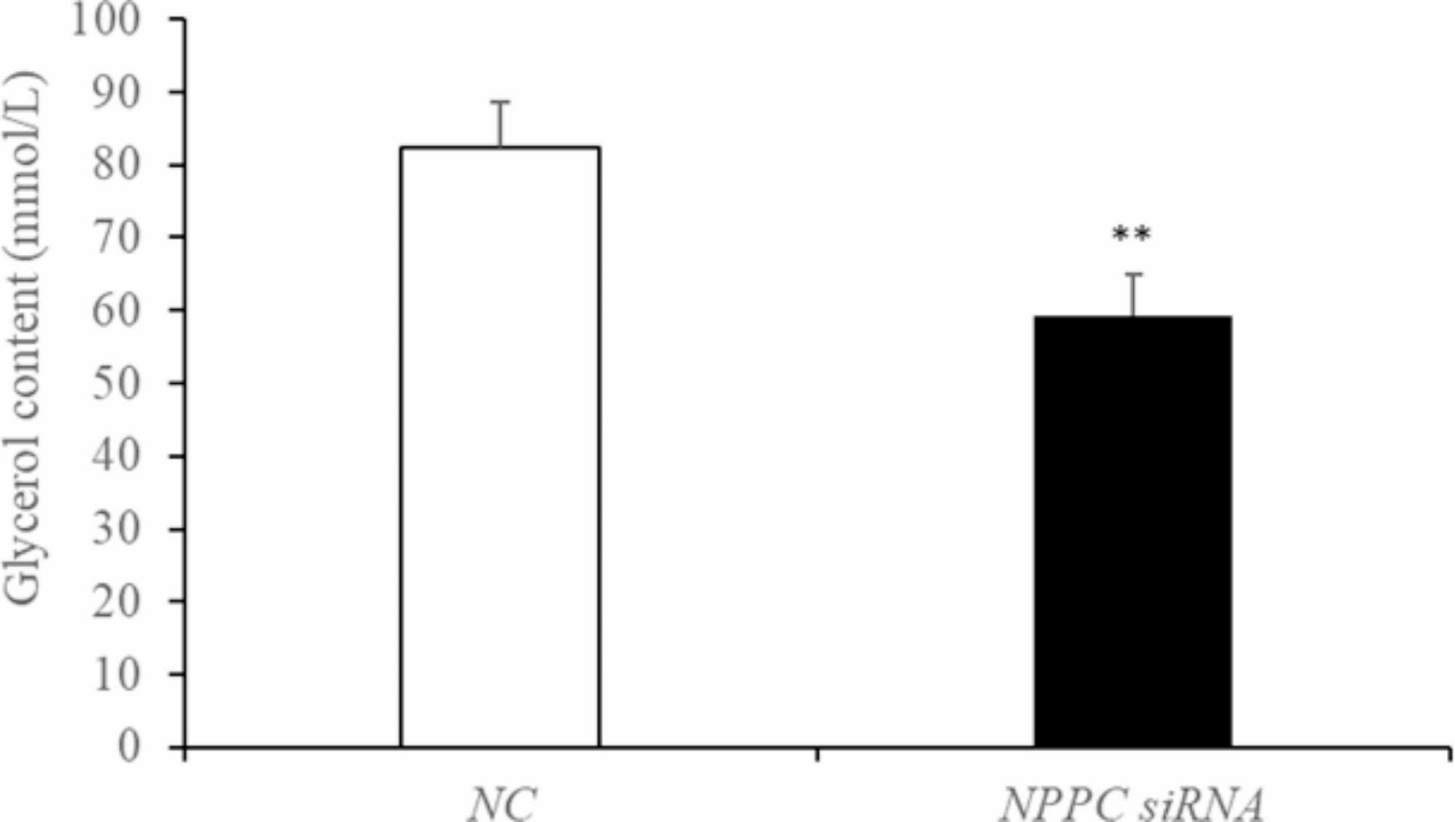
Change of the glycerol content at 4 d after transfection with *NPPC* siRNA in intramuscular adipocytes. (*n* = 3), (**p < 0.01 vs. control).

Preadipocytes were transfected with either the *NPPC* siRNA or the negative control vector. The expression of *NPPC* mRNA, compared with that of the control group, decreased significantly at 24 h after transfection with *NPPC* siRNA (Fig. 5, *p* < 0.01). The effects of *NPPC* interference on the proliferation of preadipocytes and their differentiation into adipocytes are shown in Figs. 6 and 7, respectively. The number of cells was significantly reduced at both 24 and 72 h after *NPPC* interference (Fig. 6, *p* < 0.05). Oil Red O staining showed that the lipid droplets were slightly larger, and their numbers were increased in groups treated with *NPPC* siRNA compared with those in control groups (Fig. 7B). *NPPC* interference significantly reduced glycerol levels compared with those in control cultures at 4 d (Fig. 8, *p* < 0.05).

### **Molecular mechanism of CNP regulation of IMF lipid deposition**

**Fig. 9:**
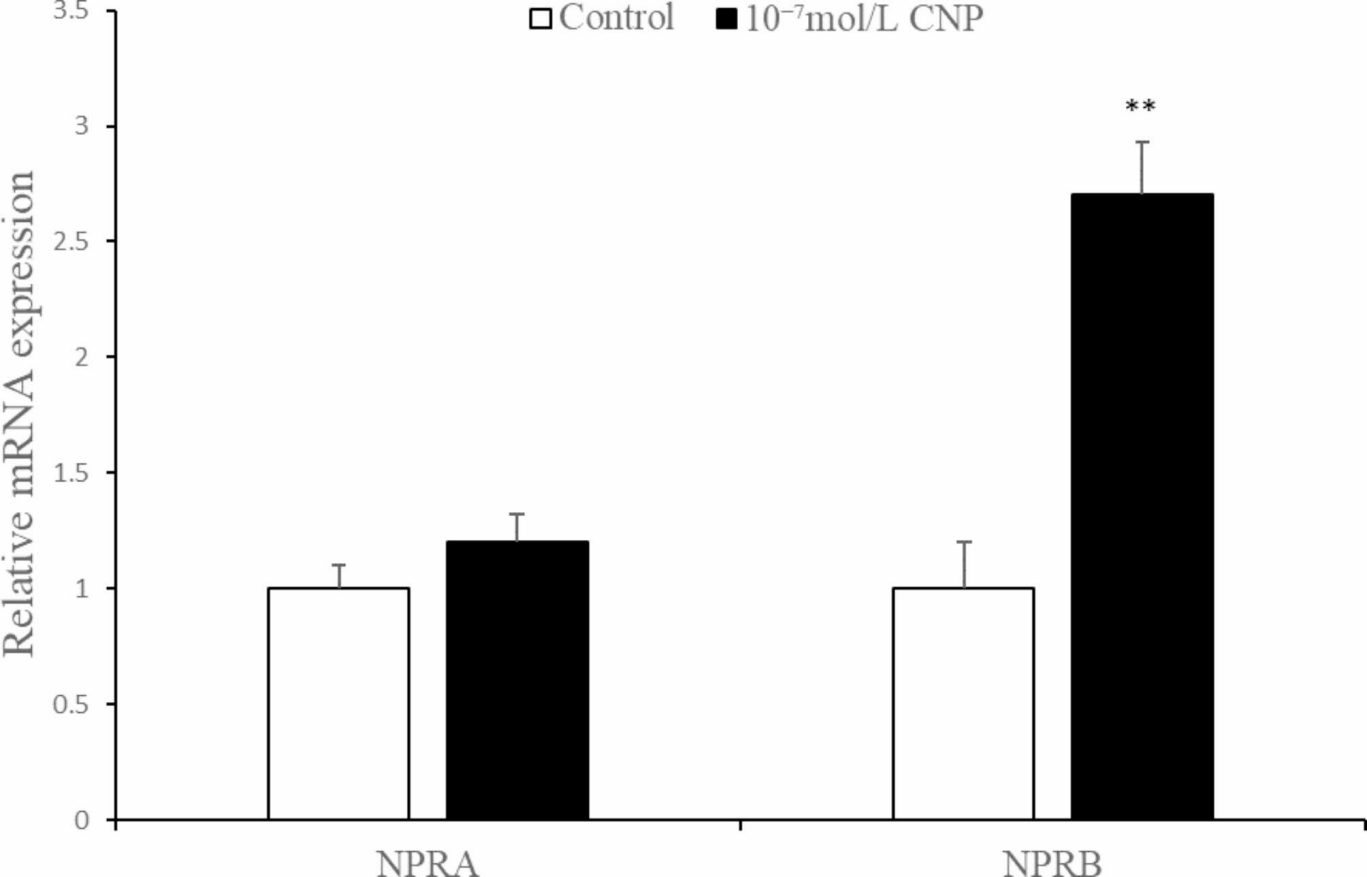
Change of *NPRB* expression in intramuscular adipocytes at 6 d after CNP treatment ( *n* = 3), (*p < 0.05 and **p < 0.01 vs. control).

**CNP promoted *NPRB*mRNA expression in IMF adipocytes   ** As shown in Fig. 9, treatment with exogenous 10^−7^ mol/L CNP for 6 d significantly increased the expression of *NPRB* mRNA (*p* < 0.05). The difference in *NPRA* expression in treated compared with control cells was not significant (*p* > 0.05).

### Key genes and pathways responding to CNP regulation in IMF adipocytes

**Fig. 10:**
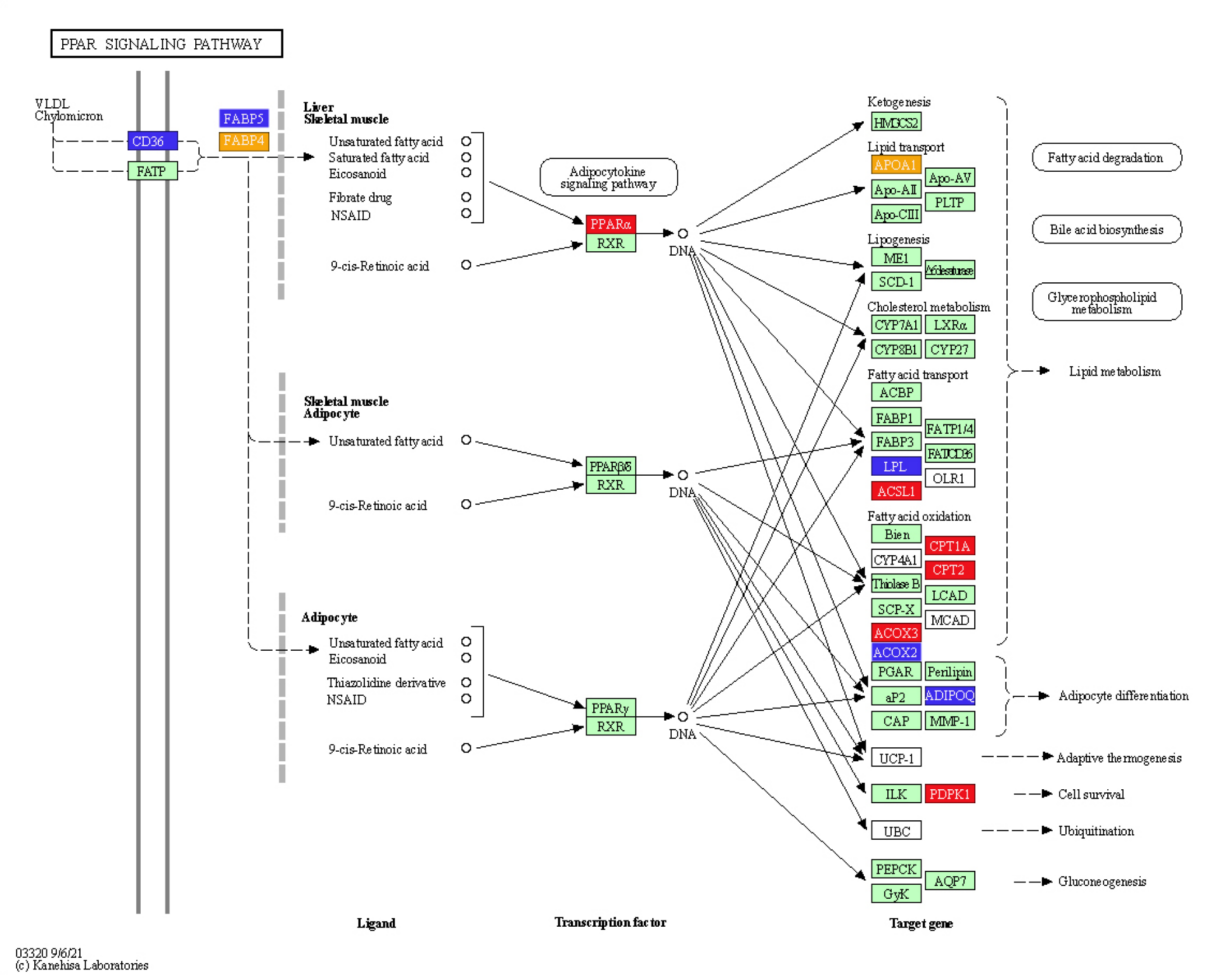
PPAR pathway from the KEGG database^44–46^.

**Fig. 11:**
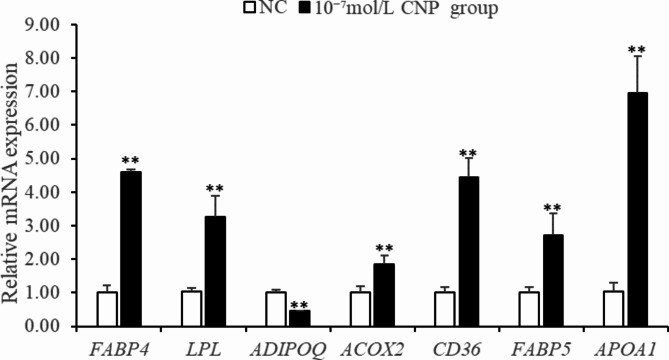
Validation of differentially expressed genes by qPCR (*n* = 3), (**p < 0.01).

mRNA sequencing was used to identify key genes and pathways by which CNP influenced lipid metabolism in IMF adipocytes. DEGs were detected in total RNA isolated from cells treated with 10^−7^ mol/L CNP and control cells not exposed to CNP. The quality control data are shown in Table S1. A total of 665 DEGs (*p* < 0.05; log > 1.5 or < 0.67) were identified; 323 were upregulated and 342 were downregulated (Table S2). Kyoto Encyclopedia of Genes and Genomes (KEGG) pathway analysis of the DEGs found 11 pathways that were significantly enriched (Table 3, *p* ≤ 0.01). They were the PPAR signaling pathway, calcium signaling pathway, adrenergic signaling in cardiomyocytes, focal adhesion, cardiac muscle contraction, phosphatidylinositol signaling system, neuroactive ligand-receptor interaction, regulation of actin cytoskeleton, extracellular matrix-receptor interaction, AGE-RAGE signaling pathway in diabetic complications, and cell adhesion molecules. Seven genes (*FABP4*, *APOA*, *ACOX2*, *ADIPOQ*, *CD36*, *FABP5*and *LPL*) enriched in the PPAR pathway were reported to be closely related to lipid metabolism (Fig. 10; Table 3). qPCR verified seven of the DEGs identified by deep sequencing, and the RNA amplified from cells treated with 0 and 10^−7^ mol/L CNP validated the gene expression profile analysis. There was consistency between the qPCR assays and the deep sequencing analysis in terms of the direction of regulation and statistical significance (Fig. 11, *p* < 0.05). Therefore, it was hypothesized that CNP regulated lipid metabolism in IMF adipocytes via the *NPRB*-PPAR pathway and by promoting the expression of genes (e.g., *FABP4*, *APOA*, *ACOX2*, *ADIPOQ*, *CD36*, *FABP5* and *LPL*) enriched in the PPAR pathway.

**Table 3:** Significantly enriched pathways for differentially expressed genes in intramuscular adipocytes.

Pathway	Enriched genes	*P*–value
Calcium signaling pathway	ADCY2,ADRA1D, ATP2B2,CACNA1H, CAMK1D, CAMK2A, CAMK4,CASQ2,CD38,CXCR4,HTR2B, HTR4,ip3ka, ITPR3,NCX1,PLCD4,TBXA2R, TNNC1,TNNC2	0.000
Adrenergic signaling in cardiomyocytes	ACTC1,ADCY2,ADRA1D, ATP1B1,ATP2B2,BCL2,CAMK2A, CREB5,Myl2,MYL3,MYL4,NCX1,TNNC1,TNNT2	0.000
Focal adhesion	ACTB, BCL2,CAV3,CHAD, fn1,IGF1,ITGA1,ITGA9,LAMA5,Myl2,MYL9,PDGFB, PGF, PPP1R12B, SHC4,THBS1, VTN	0.000
Cardiac muscle contraction	ACTC1,ATP1B1,CASQ2,Myl2,MYL3,MYL4,NCX1,TNNC1,TNNT2, UQCR11	0.000
Phosphatidylinositol signaling system	DGKB, DGKG, DGKQ, INPP5J, ip3ka, IP6K3,ITPK1,ITPR3,MTMR1,PIK3C2A, PLCD4	0.000
Neuroactive ligand-receptor interaction	ADM, ADRA1D, CHRM4,CHRNA1,CHRND, CHRNG, CNR1,CRHR2,EDN1,EDN2,EP4,GIPR, GRID1,GRM3,HTR2B, HTR4,PENK, S1PR3,SS2R, TBXA2R, VPAC2	0.001
Regulation of actin cytoskeleton	ACTB, APC2,ARHGEF4,CHRM4,CXCR4,FGF7,Fgf9,fn1,IQGAP2,ITGA1,ITGA9,Myl2,MYL9,PDGFB, PPP1R12B	0.001
ECM-receptor interaction	CD36,CHAD, fn1,ITGA1,ITGA9,LAMA5,NPNT, THBS1,VTN	0.006
AGE-RAGE signaling pathway in diabetic complications	BCL2,CDKN1B, EDN1,F3,fn1,IL6,NOX1,PLCD4,SELE	0.007
Cell adhesion molecules (CAMs)	CADM3,CDH3,CDH4,CLDN1,ITGA9,NFASC, NRCAM, SELE	0.010
PPAR signaling pathway	ACOX2,ADIPOQ, APOA1,CD36,FABP4,FABP5,LPL	0.010

### Effect of CNP on lipid metabolism in SCF adipocytes

**Fig. 12:**
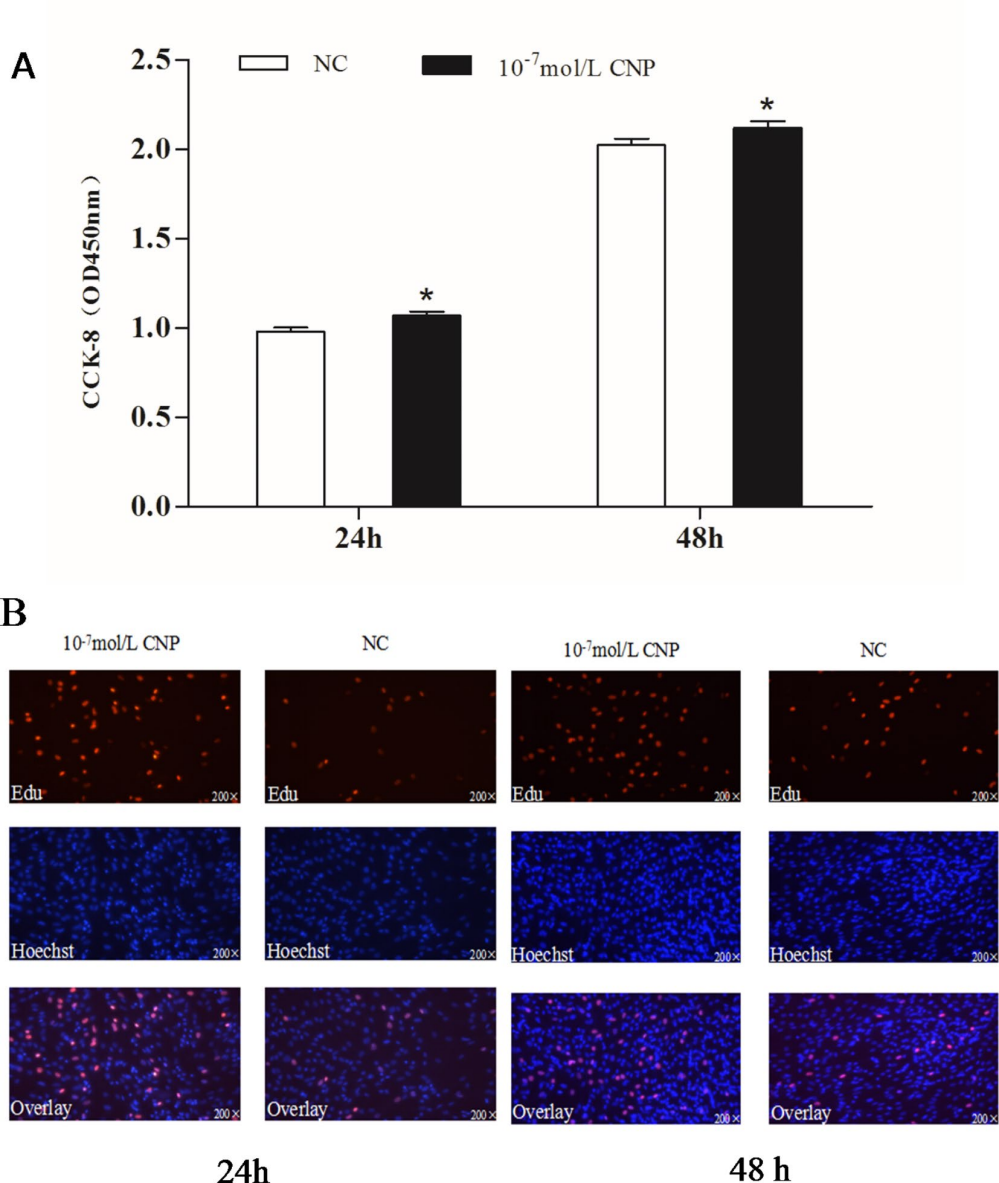
Effect of CNP on the proliferation of subcutaneous preadipocytes.(**A**) Proliferation of preadipocytes induced for 24 h and 48 h by CNP (*n* = 8, * *p* < 0.05 vs. control group). (**B**) EdU staining of intramuscular preadipocytes induced for 24 h and 72 h by CNP (*n* = 3). EdU, number of proliferative cells; Hoechst, number of cells before proliferation; Overlay, superimposition of the two images shows the location of proliferating cells. *p < 0.05 vs. control group.

**Fig. 13:**
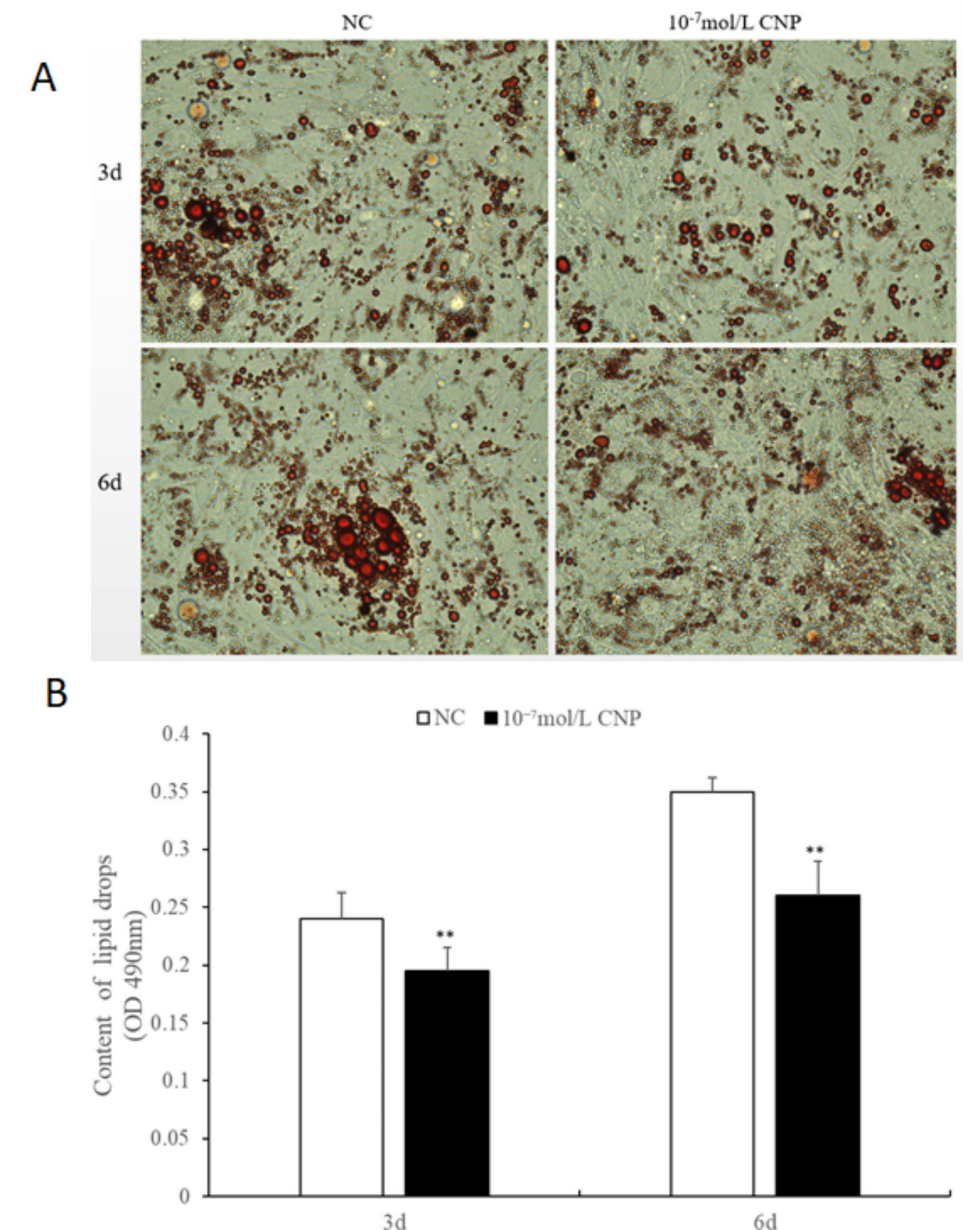
Effect of CNP on the differentiation of subcutaneous adipocytes.** A**, Morphologic changes and lipid deposition induced by 10^−7^mol/L CNP in adipocytes in vitro (inverted microscope, 200× magnification). Lipid droplets (stained with Oil Red O) were smaller and accumulated in fewer groups in cells exposed to 10^−7^mol/L CNP compared with untreated cells.** B**, Differentiation of adipocytes was significantly decreased by 10^−7^ mol/L CNP. (*n* = 3, **p < 0.01 vs. control).

**Fig. 14:**
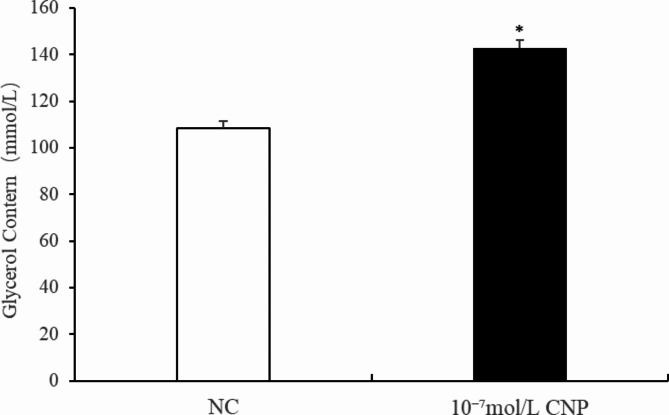
Change of the glycerol content at 6 days after CNP treatment in subcutaneous adipocytes. (*n* = 3), (*p < 0.05 vs. control).

**CNP increased preadipocyte proliferation**,** decreased adipocyte differentiation and facilitated adipocyte lipolysis** The number of preadipocytes was significantly increased by culture with 10^−7^ mol/L CNP for 24 and 48 h compared with controls (Fig. 12A, *p* < 0.05). EdU staining confirmed that the proliferation rate of CNP-induced cells was increased compared with control cells (Fig. 12B). Differentiated adipocytes were treated with exogenous 10^−7^ mol/L CNP for 3 and 6 d. Oil Red O staining showed that the lipid droplets were smaller and their numbers were decreased in study groups treated with 10^−7^ mol/L CNP compared with the control groups (Fig. 13A). The results showed that adipocyte differentiation was significantly decreased by treatment with CNP (Fig. 13B, *p* < 0.05). As shown in Fig. 14, CNP treatment significantly increased glycerol levels compared with the control group at 6 d. The results showed that CNP promoted lipolysis in SCF adipocytes.

**Fig. 15:**
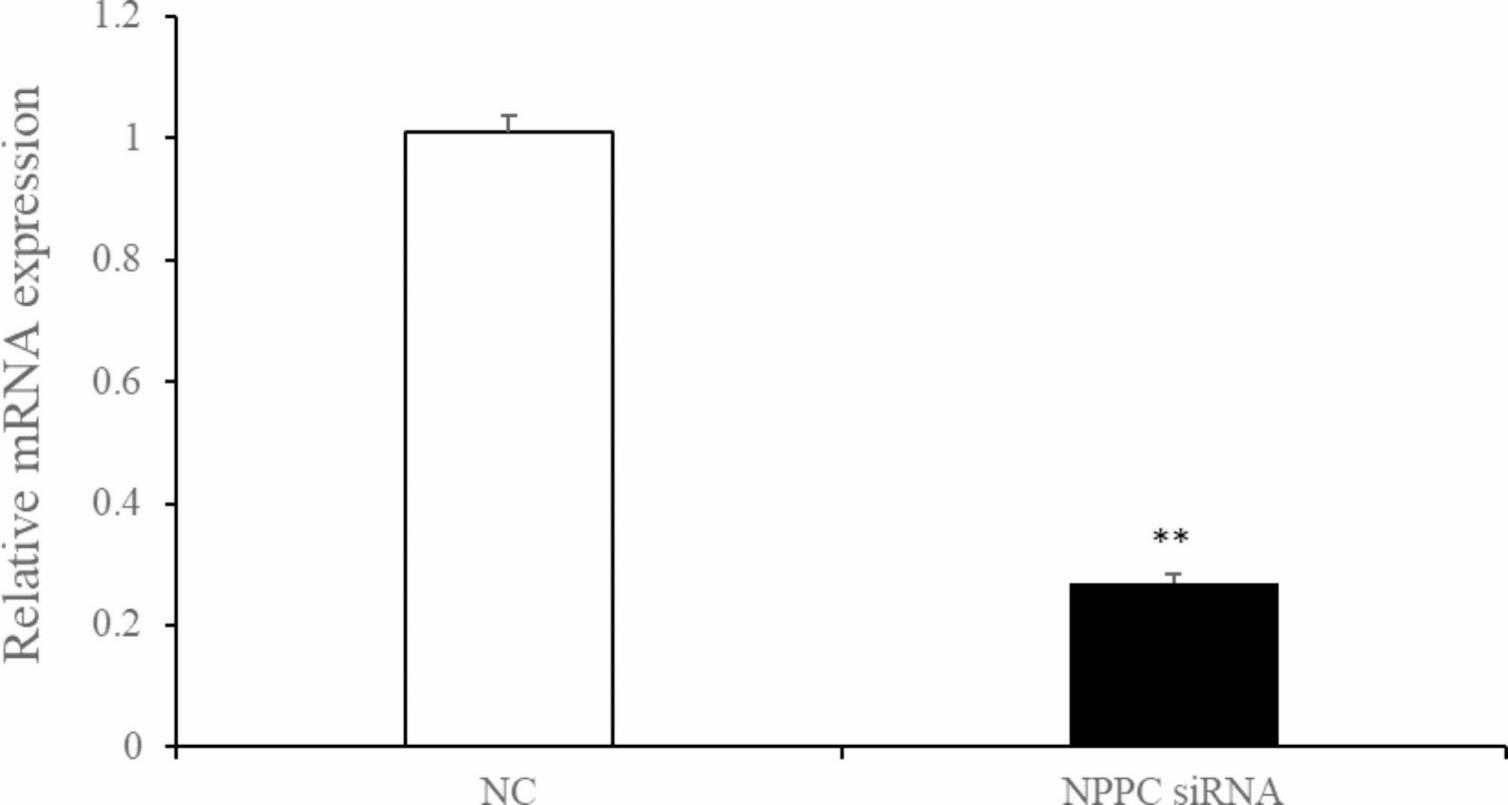
Change of *NPPC* mRNA expression in subcutaneous adipocytes after transfection with *NPPC* siRNA for 24 h. (*n* = 3) (**p < 0.01).

**Fig. 16:**
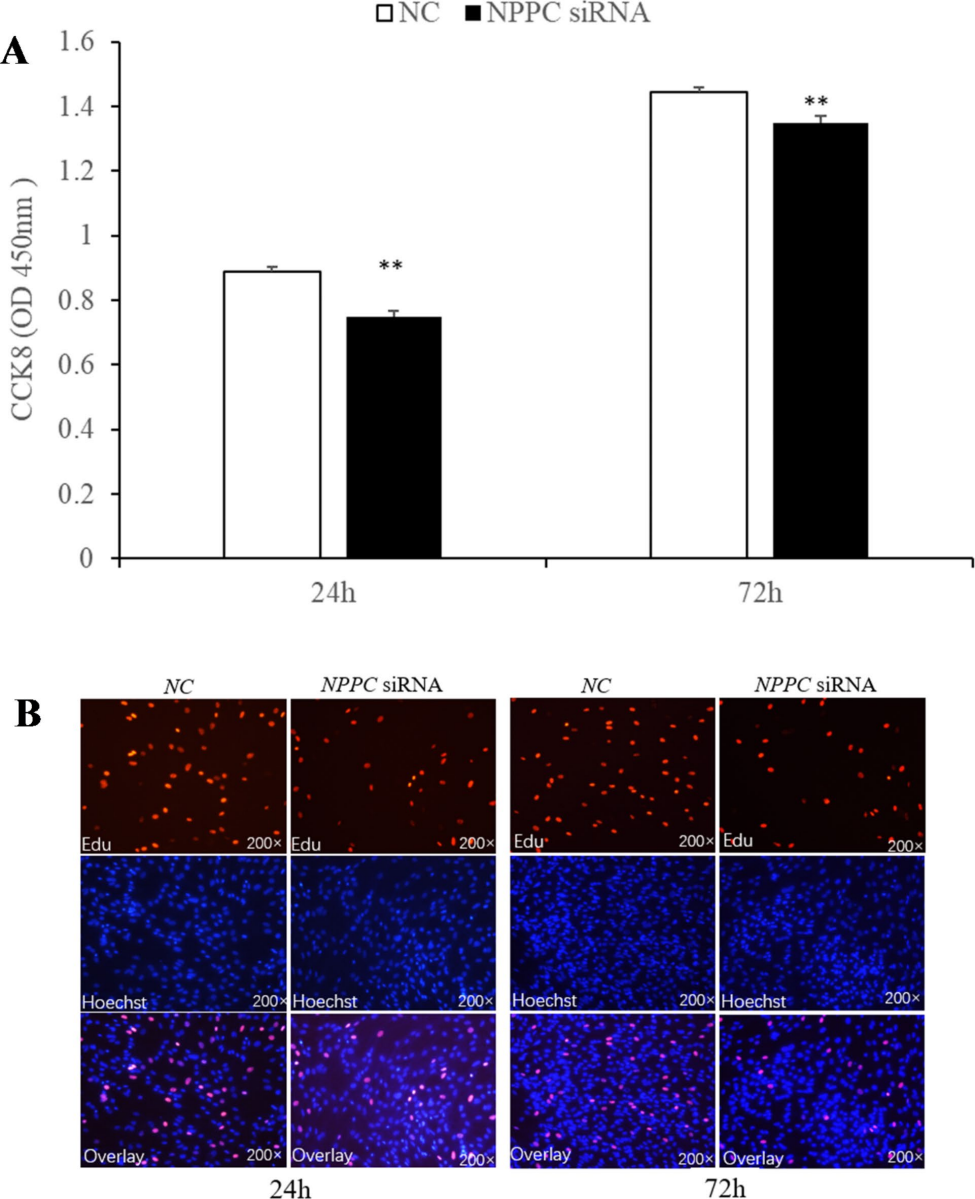
Effect of *NPPC* interference on the proliferation of subcutaneous preadipocytes (*n* = 5) (**p < 0.01 vs. control). (A) The number of preadipocytes assayed by CCK8 kits after *NPPC* interference; (B) EdU staining assay of chicken preadipocytes transfected with *NPPC* siRNA. EdU, number of proliferative cells; Hoechst, number of cells before proliferation; Overlay, superimposition of the two images shows the location of proliferating cells.

**Fig. 17:**
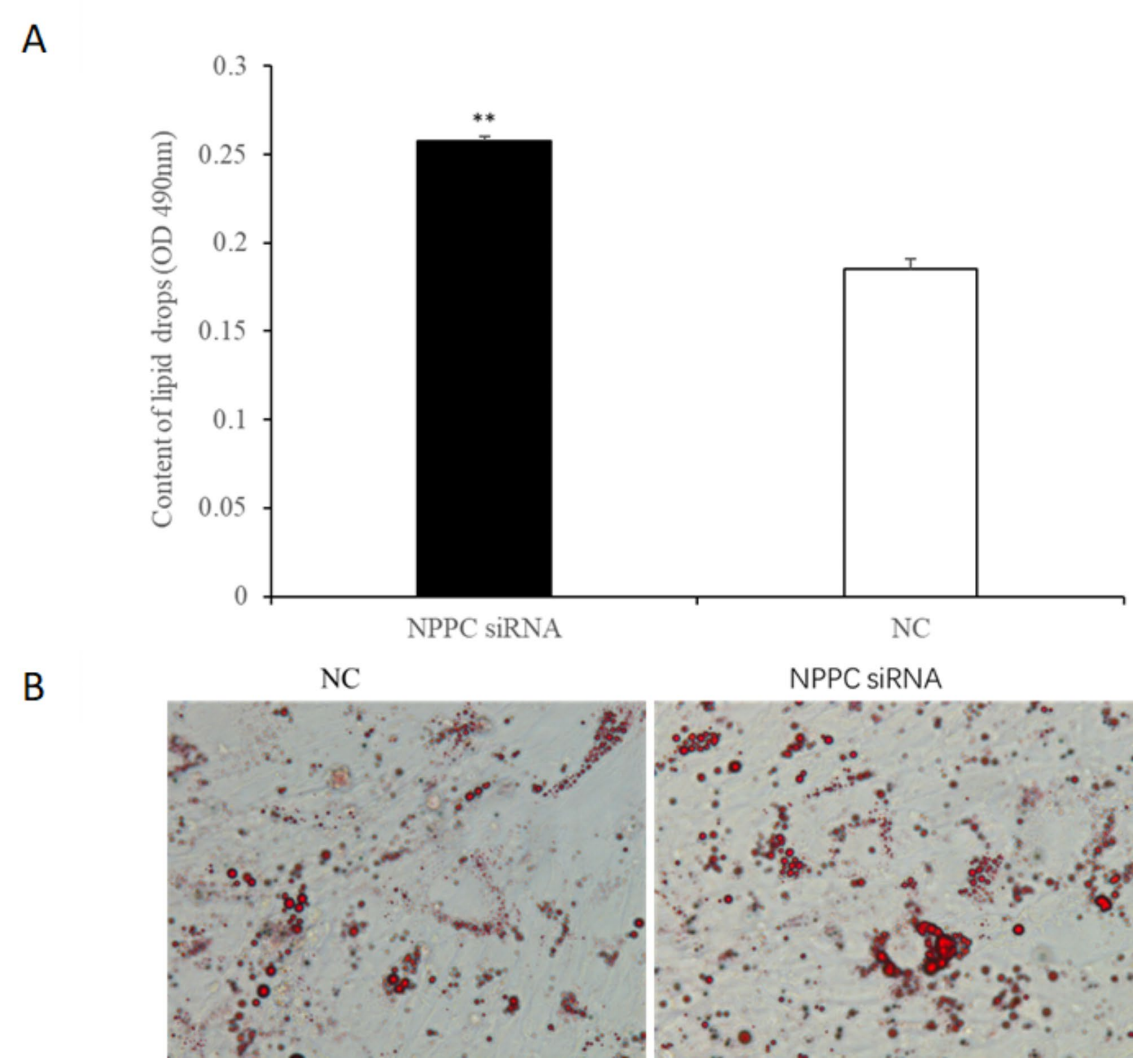
Effect of *NPPC* interference on the differentiation of subcutaneous adipocytes (**A**) The differentiation of adipocytes was significantly increased after transfection with *NPPC* siRNA (*n* = 3) (**p < 0.01 vs. control). (**B**) Morphologic changes and lipid deposition after transfection with *NPPC* siRNA in adipocytes in vitro (inverted microscope, 400× magnification). Lipid droplets (stained with Oil Red O) accumulated in greater numbers and in larger groups in cells transfected with *NPPC* siRNA compared with the negative control vector.

**Fig. 18:**
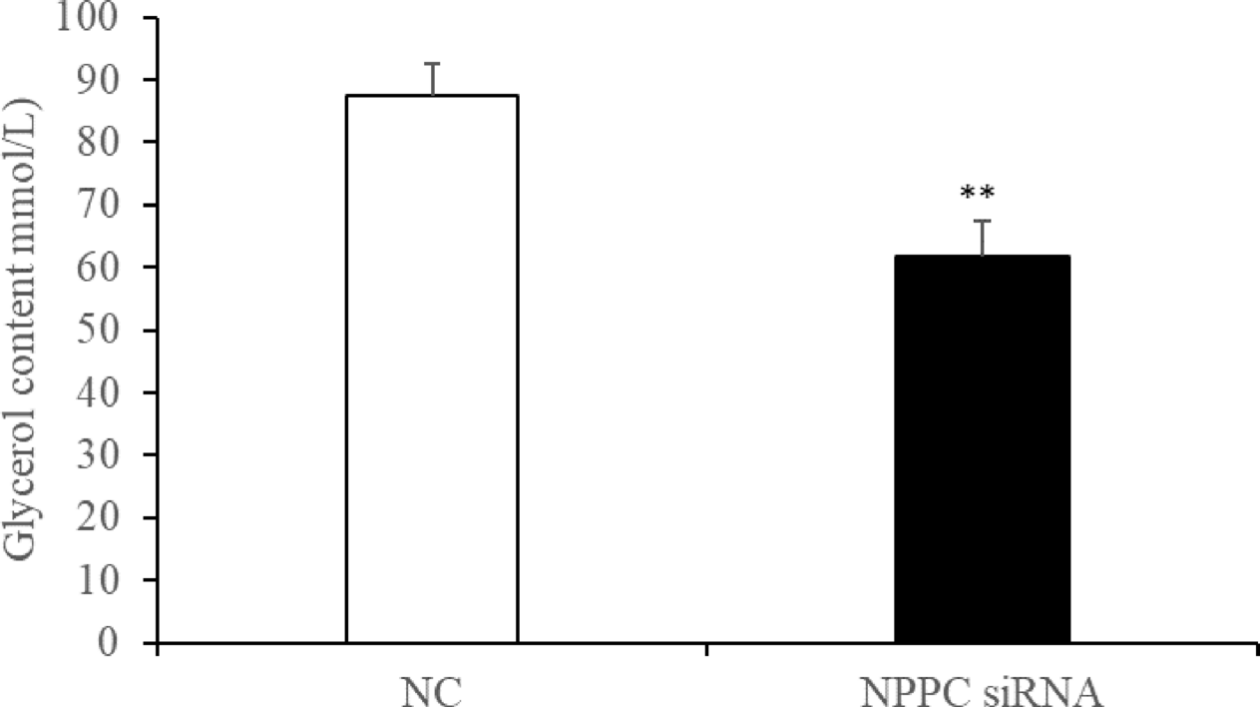
Change of the glycerol content at 4 d after transfection with *NPPC* siRNA in subcutaneous adipocytes. (*n* = 3), (**p < 0.01 vs. control).

***NPPC *interference reduced cell proliferation**,** enhanced adipocyte differentiation**,** and reduced lipolysis. ***NPPC* mRNA expression was significantly decreased compared with the control group at 24 h after transfection of preadipocytes with *NPPC* siRNA (Fig. 15, *p* < 0.05). As shown in Fig. 16, the number of cells was reduced significantly at 24 and 72 h after transfection with *NPPC* siRNA (*p* < 0.05). Lipid accumulation was increased at 3 d (Fig. 17A, *p* < 0.01). Staining with Oil Red O showed that the lipid droplets were slightly larger, and their number was increased in groups transfected with *NPPC* siRNA compared with the control groups (Fig. 17B). *NPPC* interference significantly reduced the glycerol level at 4 d compared with the control cultures (Fig. 18, *p* < 0.05).

### Molecular mechanism of CNP regulation of lipid deposition in SCF adipocytes

**Fig. 19:**
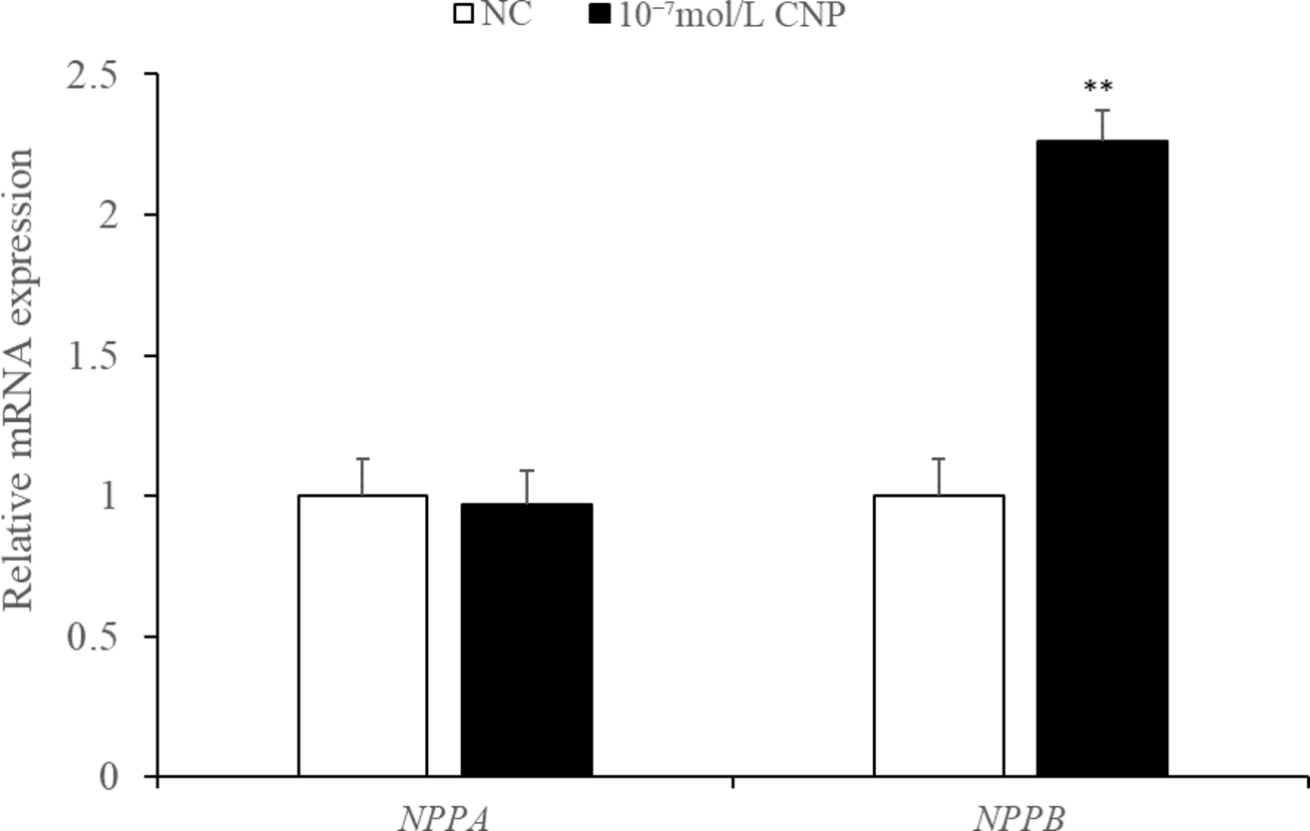
Change of *NPRB* expression in subcutaneous adipocytes at 6 d after CNP treatment ( *n* = 3), (*p < 0.05 and **p < 0.01 vs. control).

**CNP enhanced *NPRB *expression** qPCR showed that CNP treatment significantly increased the expression of *NPRB* mRNA at 6 d (Fig. 19, *p* < 0.05) and that differences in.

*NPRA* mRNA expression in treated and control cells were not significant.

### Key genes and pathways responding to CNP regulation

**Fig. 20:**
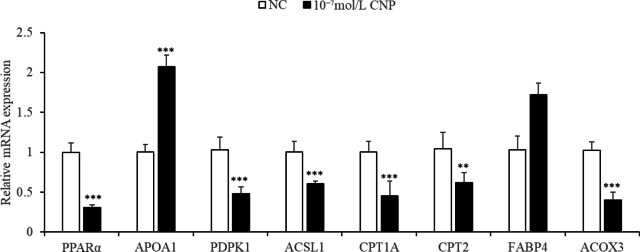
Validation of differentially expressed genes by qPCR (*n* = 3) (**p < 0.01 and ***p < 0.001).

Key DEGs and pathways were detected by mRNA sequencing of total RNA extracted from SCF adipocytes treated with 10^−7^ mol/L CNP and from control cells. The quality control data are shown in Table S1. A total of 991 DEGs (*p* < 0.05; log 1.5 > 1.5 or < 0.67) were identified, with 136 upregulated genes and 855 downregulated genes (Table S3). After KEGG pathway analysis of the DEGs, 12 pathways were significantly enriched (Table 4, *p* ≤ 0.01). They were PPAR signaling, ubiquitin mediated proteolysis, animal autophagy, vascular smooth muscle contraction, focal adhesion, regulation of actin cytoskeleton, apoptosis, endocytosis, transforming growth factor-beta signaling, progesterone-mediated oocyte maturation, protein processing in the endoplasmic reticulum, and NOD-like receptor signaling pathway. Eight genes (*ACOX3*, *ACSL1*, *APOA1*, *CPT1A*, *CPT2*, *FABP4*, *PDPK1* and *PPARα*) enriched in PPAR pathway were reported to be closely related to lipid metabolism. qPCR verified the consistency of the eight DEGs identified by deep sequencing in terms of the direction of regulation and statistical significance (Fig. 20., *p* < 0.05). Taken together, the results support the hypothesis that CNP regulated lipid metabolism in SCF adipocytes via the *NPRB*-PPAR pathway and by increasing the expression of genes (e.g., *ACOX3*, *ACSL1*, *APOA1*, *CPT1A*, *CPT2*, *FABP4*, *PDPK1* and *PPARα*) enriched in the PPAR pathway.

**Table 4:** Significantly enriched pathways of differentially expressed genes in subcutaneous adipocytes.

Pathway	Enriched genes	*P*-value
Ubiquitin mediated proteolysis	BIRC6,CUL1,CUL4A, CUL4B, CUL5,HERC1,HERC2,IAP3,ITCH, NEDD4,TRIP12,UBA6,UBE2L3,UBE3C, ANAPC4	0.007
Autophagy - animal	ATG13,ATG2B, CFLAR, EIF2AK4,MAPK1,MAPK9,Metazoa_SRP, PDPK1,PIK3C3 PIK3CA, PIK3CB, PRKAA1,PRKCQ, RB1CC1,RRAGD	0.009
Vascular smooth muscle contraction	ADCY2,CALCRL, CALD1,cRhoA, GUCY1B3,MAPK1,MBSP, MRVI1,MYLK, PLA2G2E, PLA2G4A PRKCH, PRKCQ, ROCK1, ROCK2	0.009
Focal adhesion	ARHGAP5,COL1A2,cRhoA, IAP3,ITGA2,ITGA4,ITGAV, ITGB1,ITGB8,MAPK1,MAPK9,MBSP, MYLK, PARVA, PDPK1,PIK3CA, PIK3CB, ROCK1,ROCK2, VTN,	0.009
Regulation of actin cytoskeleton	APC, ARHGEF4,BAIAP2,BDKRB1,cRhoA, DIAPH1,Fgf9,ITGA2,ITGA4,ITGAV, ITGB1,ITGB8,MAPK1,MBSP, MYLK, PIK3CA, PIK3CB, ROCK1,ROCK2, SCIN	0.010
Apoptosis	SPTAN1,PIK3CB, PIK3CA, PDPK1,MAPK9,MAPK1,IAP3,CTSZ, CTSO, CFLAR, CASP3,CASP2,ATM APAF1	0.031
Endocytosis	AP2B1,ARFGEF2,CAPZA1,cRhoA, DAB2,DNM3,EEA1,EHD4,IGF2R, IQSEC1,ITCH, KIF5B, NEDD4 RAB11FIP2,RABEP1,RBSN, STAM, TFRC, VPS29, VPS4B	0.040
TGF-beta signaling	THSD4,ROCK1,MAPK1,INHBA, DCN, CUL1,cRhoA, CHRD, BMPR-II, BMPR1A, AMH	0.041
PPAR signaling	ACOX3,ACSL1,APOA1,CPT1A, CPT2,FABP4,HMGCS1,PDPK1,PPARA	0.043
Progesterone-mediated oocyte maturation	ADCY2,ANAPC4,BUB1,HSP90AA1, MAPK1, MAPK9,PIK3CA, PIK3CB, RPS6KA3,RPS6KA6	0.045
Protein processing in endoplasmic reticulum	CUL1,DNAJC5,EIF2AK4,HSP90AA1,HSP90B1,HSPA4L, MAPK9,PDIA4,Sect. 62,SEL1L, TRAM1 UGGT2,WFS1,YOD1, ATXN3	0.048
NOD-like receptor signaling	ANTXRL, cRhoA, ERBB2IP, HSP90AA1, IAP3, IFNAR1, MAPK1, MAPK9TANK, TBK1, TP53BP1, TRAF3, TRPM7	0.050

### Integrated analysis of DEGs from IMF adipocytes and SCF adipocytes

**Fig. 21:**
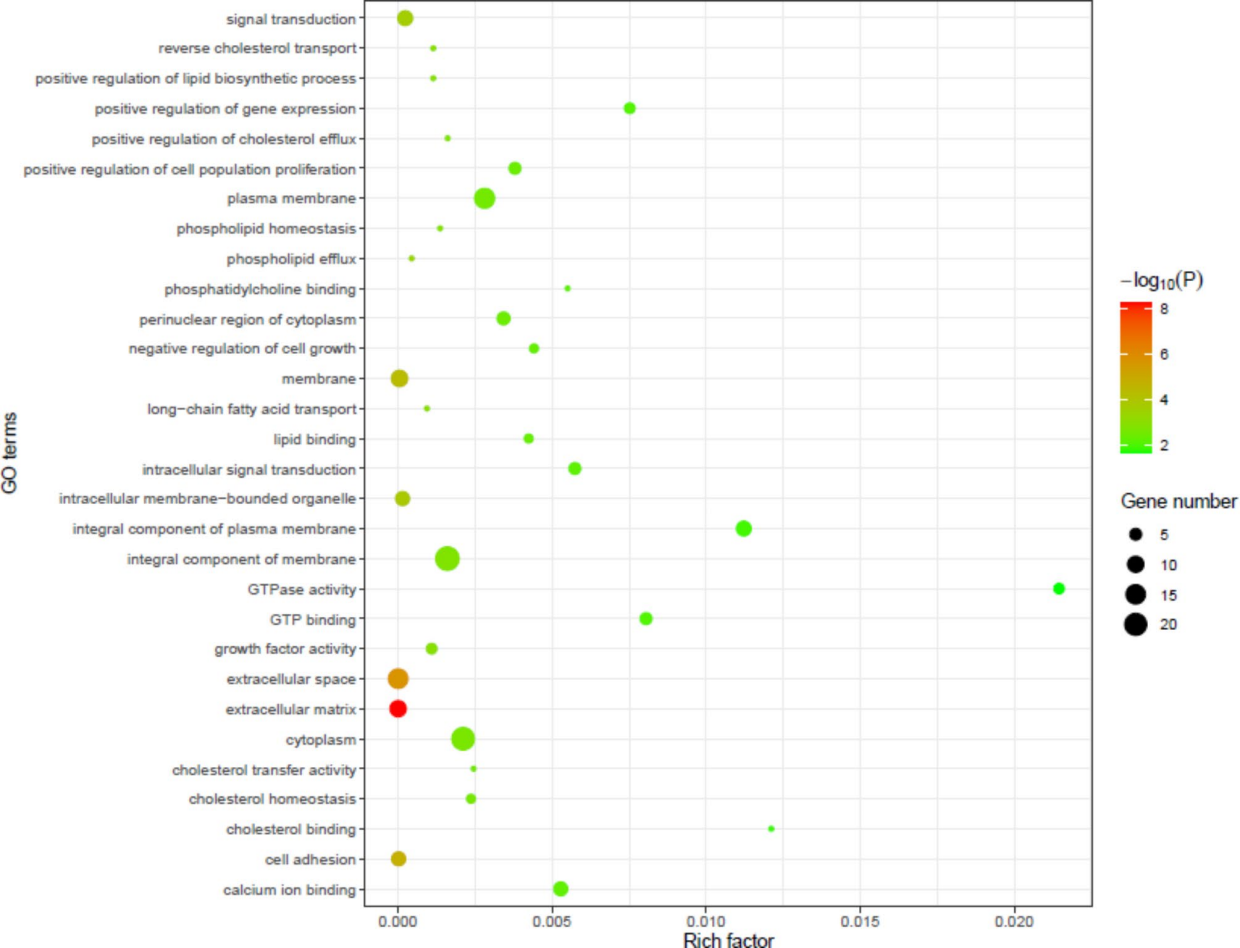
GO analysis of intersection genes in both IMF and SCF adipocytes.

**Fig. 22:**
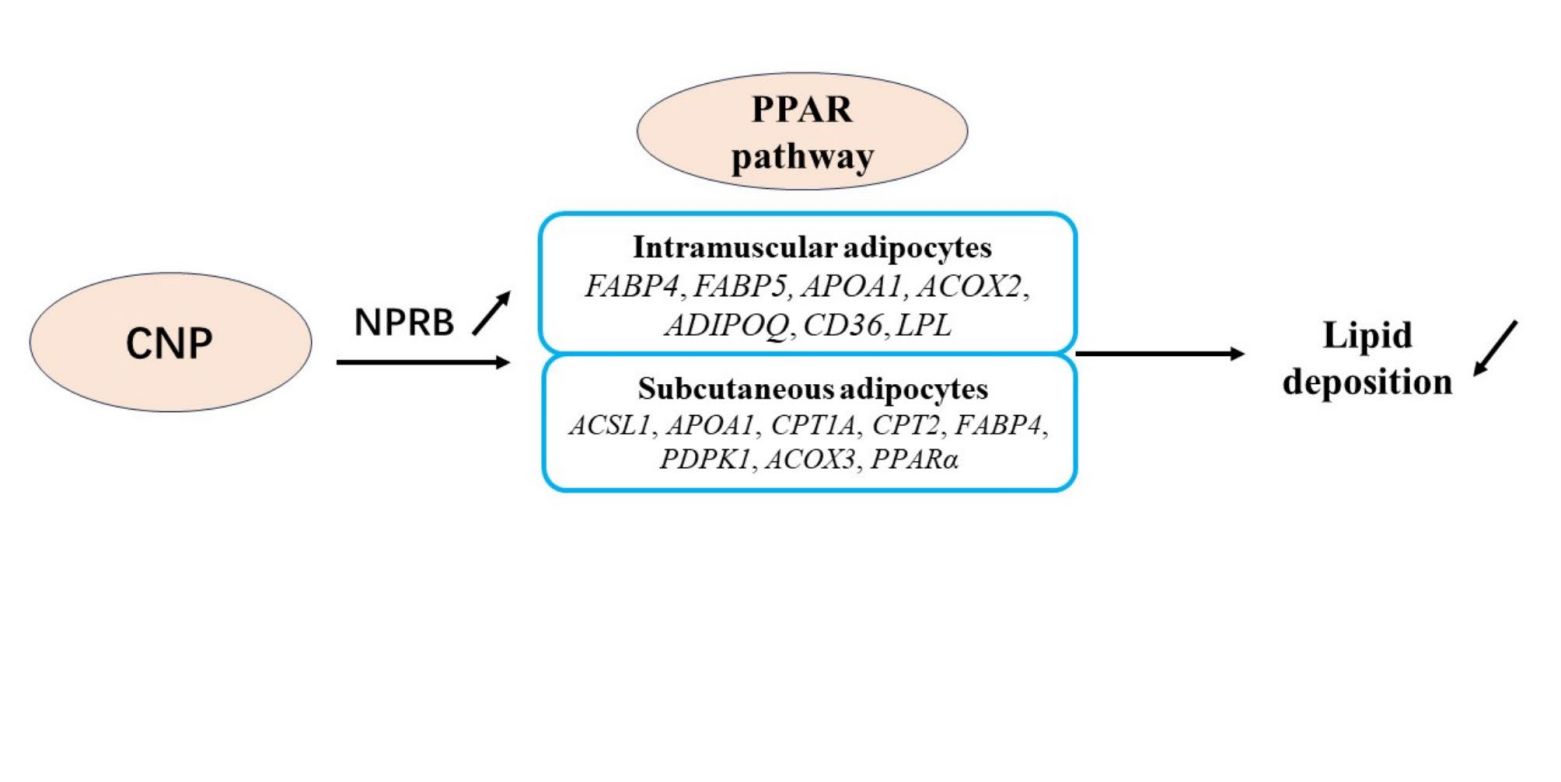
CNP regulated lipid metabolism through the *NPRB*-PPAR pathway in both IMF and SCF adipocytes. In IMF adipocytes, metabolism was regulated mainly by *FABP4*, *FABP5*, *APOA1*, *ACOX2*, *ADIPOQ*, *CD36*, and *LPL* enriched PPAR pathways, and by *ACSL1*, *APOA1*, *CPT1A*, *CPT2*, *FABP4*, *PDPK1*, *ACOX3*, and PPARα enriched PPAR pathways in SCF adipocytes.

A total of 665 DEGs were identified in IMF adipocytes and 991 in SCF adipocytes. To further understand the effect of those DEGs on lipid deposition of IMF and SCF adipocytes, an integrated analysis was performed, and found 113 “intersection genes” that were common to both IMF and SCF adipocytes (Table S4). A total of 124 GO terms (gene number ≥ 2, *p* < 0.05) were significantly enriched based on 113 intersection genes, including integral component of membrane, cytoplasm, plasma membrane, extracellular space, signal transduction, cell adhesion, calcium ion binding, positive regulation of cell population proliferation, intracellular signal transduction, GTP binding, growth factor activity, positive regulation of gene expression, GTPase activity, cholesterol homeostasis, lipid binding, etc. The GO terms related to cell differentiation and lipid metabolism are shown in Fig. 21. KEGG pathway analysis of the intersection genes found that the significantly enriched pathways (Table 5) included PPAR signaling, tumor growth factor-beta signaling, calcium signaling, cytokine-cytokine receptor interaction, apelin signaling, adrenergic signaling in cardiomyocytes and cell adhesion molecules. It is noteworthy that the PPAR pathway was significantly enriched not only in intersection genes, but also in the DEGs in both IMF and SCF adipocytes. Two intersection genes, *APOA1* and *FABP4*, were enriched in the PPAR pathway. The results suggest that CNP regulated lipid metabolism of IMF and SCF adipocytes through different genes enriched in the PPAR pathway and that *APOA1* and *FABP4* may be key genes (Fig. 22).

**Table 5:** Pathways significantly enriched in intersection genes.

Pathway	Enriched genes	*p*-value
Cell adhesion molecules (CAMs)	CADM1,CADM3,CDH4,SELE	0.003
TGF-beta signaling pathway	AMH, INHBA, THSD4	0.009
Calcium signaling pathway	ADCY2,ATP2B2,CAMK4,HTR2B	0.011
Cytokine-cytokine receptor interaction	AMH, CCR7, INHBA, TNFRSF11B	0.016
Apelin signaling pathway	ADCY2,MYL4,CAMK4	0.019
Adrenergic signaling in cardiomyocytes	ADCY2,ATP2B2,MYL4	0.023
PPAR signaling pathway	APOA1, FABP4	0.037

## Discussion

### CNP and lipid metabolism in IMF adipocytes

Addition of 10^−7^ mol/L CNP to IMF adipocytes significantly decreased differentiation and increased cell proliferation and glycerin level. The findings clearly support the hypothesis that CNP played an important role in regulating lipid metabolism in the skeletal muscle of chickens. Addition of CNP to cultured IMF adipocytes significantly upregulated the expression of *NPRB* mRNA but had no effect on *NPRA*. Previous studies demonstrated that ANP stimulated lipolysis by binding to its receptor *NPRA*in both human and rat adipocytes^24,25^. CNP acts by binding to its receptor, NPRB^26,27^. The overexpression of CNP in adipocytes has been shown to decrease adipocyte hypertrophy via upregulation of *NPRB*in white adipose tissue^28^. mRNA sequencing identified seven DEGs (*FABP4*, *FABP5*, *APOA1*, *ACOX2*, *ADIPOQ*, *CD36*, and *LPL*) in adipocytes that are known to be linked to lipid metabolism and were found by KEGG analysis to be significantly enriched in the PPAR pathway. Fatty acid-binding protein 4 (*FABP4*) and fatty acid-binding protein 5 (*FABP5*) are cytoplasmic proteins involved in glucose and lipid metabolism^29^. *FABP5*interacts with numerous long-chain fatty acids of varying degrees of saturation, and participates in cis-bonded, polyunsaturated fatty-acid signaling^30^. High-density lipoprotein was significantly increased in obese (ob/ob) mice with a defect in the hepatic catabolism of apolipoprotein A1 (*APOA1)*^31^. CD36 was reported to promote fatty-acid uptake by muscle and fat cells^32^. Lipoprotein lipase (LPL) is the rate-limiting enzyme for the hydrolysis of the triglyceride core of circulating TG-rich lipoproteins, chylomicrons, and VLDLs^33^. Adiponectin (ADIPOQ) is a protein hormone secreted by adipocytes and regulates lipid and carbohydrate metabolism^34^, including promotion of fatty acid oxidation, decrease of plasma triglycerides, improvement of glucose metabolism and increased insulin sensitivity^35^. Of three Acyl-CoA oxidases (ACOXs), ACOX1, ACOX2, and ACOX3, *ACOX2*is a highly expressed regulatory gene in fatty acid metabolism pathways^36^. The results obtained here revealed that CNP bound with *NPRB* and upregulated its expression. It then changed the expression pattern of seven genes (*FABP4*, *FABP5*, *APOA1*, *ACOX2*, *ADIPOQ*, *CD36*, and *LPL*), which ultimately resulted in altered IMF adipocyte differentiation and lipolysis.

### CNP and lipid metabolism in SCF adipocytes

Addition of 10^−7^ mol/L CNP to SCF adipocytes significantly decreased differentiation and increased proliferation and glycerin concentration. The results confirmed that CNP regulated lipid metabolism in not only IMF, but also in SCF adipocytes. More important, *NPRB* mRNA expression and the cGMP level were significantly increased, which is consistent with the trend in IMF adipocytes. Specifically, the PPAR pathway was also significantly enriched, but the enriched genes in SCF adipocytes were different from those in IMF adipocytes except for *APOA1* and *FABP4*. Eight of the genes (*ACSL1*, *APOA1*, *CPT1A*, *CPT2*, *FABP4*, *PDPK1*, *ACOX3* and *PPARα*) are known to be linked to lipid metabolism. Acyl-CoA synthetase long-chain family member 1 (*ACSL1)*influences lipolysis^37^and the β-oxidation rate in 3T3-L1 adipocytes^38^. The microRNA gga-miR-19b-3p was shown to accelerate adipocyte lipid deposition by downregulating *ACSL1*in chickens^19^. Peroxisome proliferator-activated receptor alpha (*PPARα*) is highly expressed in liver and muscle tissues, where it contributes to the β-oxidation of fatty acids and reduces lipid accumulation^39^. In chickens, PPARα mRNA expression was found to be negatively correlated with IMF from 0 to 8 w in thigh muscle tissue^40^. Carnitine palmitoyl transferase 2 (CPT2) is an enzyme required for mitochondrial long-chain fatty acid oxidation, and the loss of muscle *CPT2*led to accumulation of long-chain acylcarnitine and protected against diet-induced obesity and insulin resistance in mice^41^; Mitochondrial carnitine palmitoyl transferase 1a (CPT1A) in the liver mitochondrial outer membrane was found to catalyze the primary regulatory step in overall mitochondrial fatty acid oxidation^42^. *ACOX3*is involved in fatty acid metabolism^43^. The 113 intersection genes were included in six significantly enriched pathways. It is interesting that the PPAR pathway was also significantly enriched, including *APOA1* and *FABP4*, suggesting that the PPAR pathway is the key pathway in CNP-regulated lipid metabolism in both IMF and SCF adipocytes, *APOA1* and *FABP4* may be the key genes.

In summary, our data showed that CNP regulated lipid metabolism through the NPRB-cGMP-PPAR pathway in both IMF and SCF adipocytes. In IMF adipocytes, metabolism was regulated mainly by *FABP4*, *FABP5*, *APOA1*, *ACOX2*, *ADIPOQ*, *CD36*, and *LPL* enriched PPAR pathways, and by *ACSL1*, *APOA1*, *CPT1A*, *CPT2*, *FABP4*, *PDPK1*, *ACOX3*, and PPARα enriched PPAR pathways in SCF adipocytes. *APOA1* and *FABP4* were key genes.

## Electronic supplementary material

Below is the link to the electronic supplementary material.

Supplementary Material 1

## Data Availability

All data generated or analysed during this study are included in this published article [and its supplementary information files].

## Abbreviations

AbF: Abdominal Fat
ANP: Atrial Natriuretic Peptide
BNP: Brain Natriuretic Peptide
CCK-8: Cell Counting Kit-8
CNP: Intramuscular Fat
DEGs: Differentially Expressed Genes
DMEM: Dulbecco’s Modified Eagle Medium
EdU: 5-ethynyl-2’-deoxyuridine
FPKM: Fragments per Kilobase of Exon per Million Mapped Reads
FSH: Follicle Stimulating Hormone
IMF: Subcutaneous Fat
KEGG: Kyoto Encyclopedia of Genes and Genomes
LPL: Lipoprotein Lipase
NPPC: Natriuretic Peptide C
NPRA: Natriuretic Peptide Receptor A
NPRB: Natriuretic Peptide Receptor B
NPS: Natriuretic Peptides
PPAR: Peroxisome Proliferator Activated Receptor
qPCR: Quantitative Real-Time Polymerase Chain Reaction
SCF: C-Type Natriuretic Peptide
VLDL: Very Low-Density Lipoprotein
